# Supplementary material for: Persimmon Proanthocyanidins with Different Degrees of Polymerization Possess Distinct Activities in Models of High Fat Diet Induced Obesity
Source: Nutrients. 2022 Sep 9;14(18):3718. doi: 10.3390/nu14183718 (PMC9505881; doi:10.3390/nu14183718)
Supplement: Supplementary file 1 [file nutrients-14-03718-s001.zip › nutrients-1861413-supplementary.pdf]

Supplementary Table S1. The formula of D12450B and D12492.

**Product #**	**D12450B**		D12492	
	**10 kcal% Fat**		60 kcal% Fat	
	gm%	kcal%	gm%	kcal%
Protein	16.9	20.0	26.2	20.0
Carbohydrate	67.3	70.0	26.3	20.1
Fat	4.3	10.0	34.9	59.9
kcal/gm	3.85		5.24	
Ingredient	gm	kcal	gm	kcal
Casein	200	800	200	800
L-Cystine	3	12	3	12
Corn Starch	506.2	2024.8	0	0
Maltodextrin 10	125	500	125	500
Sucrose	68.8	275.2	68.8	275.2
Cellulose, BW200	50	0	50	0
Soybean Oil	25	225	25	225
Lard	20	180	245	2205
Mineral Mix S10026	10	0	10	0
DiCalcium Phosphate	13	0	13	0
Calcium Carbonate	5.5	0	5.5	0
Potassium Citrate, 1 H_2O	16.5	0	16.5	0
Vitamin Mix V10001	10	40	10	40
Choline Bitartrate	2	0	2	0
FD&C Yellow Dye #5	0.04	0	0	0
FD&C Blue Dye #1	0.01	0	0.05	0
FD&C Blue Dye #1	0.01	0	0.05	0
